# Making Memories: The Development of Long-Term Visual Knowledge in Children with Visual Agnosia

**DOI:** 10.1155/2013/306432

**Published:** 2013-11-10

**Authors:** Tiziana Metitieri, Carmen Barba, Simona Pellacani, Maria Pia Viggiano, Renzo Guerrini

**Affiliations:** ^1^Pediatric Neurology Unit, Children's Hospital A. Meyer, University of Florence, Viale Pieraccini 24, 50139 Firenze, Italy; ^2^IRCCS Stella Maris, Viale del Tirreno 331, Calambrone, 56018 Pisa, Italy; ^3^Department of Neuroscience, Psychology, Pharmacology and Child Health, University of Florence, Viale Pieraccini 6, 50139 Firenze, Italy

## Abstract

There are few reports about the effects of perinatal acquired brain lesions on the development of visual perception. These studies demonstrate nonseverely impaired visual-spatial abilities and preserved visual memory. Longitudinal data analyzing the effects of compromised perceptions on long-term visual knowledge in agnosics are limited to lesions having occurred in adulthood. The study of children with focal lesions of the visual pathways provides a unique opportunity to assess the development of visual memory when perceptual input is degraded. We assessed visual recognition and visual memory in three children with lesions to the visual cortex having occurred in early infancy. We then explored the time course of visual memory impairment in two of them at 2 years and 3.7 years from the initial assessment. All children exhibited apperceptive visual agnosia and visual memory impairment. We observed a longitudinal improvement of visual memory modulated by the structural properties of objects. Our findings indicate that processing of degraded perceptions from birth results in impoverished memories. The dynamic interaction between perception and memory during development might modulate the long-term construction of visual representations, resulting in less severe impairment.

## 1. Introduction 

Visual agnosia is a modality-specific disorder of object recognition caused by a lesion involving the visual cortex [1]. As originally described by Lissauer [2] the disorder cannot be attributed to poor sensory processing, and recognition of objects through other modalities can be relatively preserved. Most of our understanding of visual agnosia derives from investigations of adults with visual agnosia acquired after years of normal functioning [3]. Previous findings suggest that long-term memory and recognition can be concurrently or differentially impaired [1]. In the majority of cases of agnosia both memory and perception are clearly impaired [4], though, in some, the representations stored in memory are preserved even for objects that cannot be recognized [5–7]. However, data from agnosics exploring the dynamic influence of degraded perceptions on the updating of long-term visual knowledge are limited. The longitudinal investigation of the profoundly agnosic H.J.A. demonstrated a subtle deterioration in his ability to draw objects from memory over time [8]. These data might suggest an interplay between perception and memory rather than their functional independence [8]: when perceptual processing is impaired, visual memory may gradually decline, due to less fine-tuning of the system to the visual properties of objects.

Of particular interest may be the observation of these dynamic changes in children with lesions which occurred around birth in whom perceptual impairments lead to weak updating of visual memories that starts early in life. Reported observations of childhood visual agnosia are scarce, and, in most of them, the causative lesion had occurred after visual recognition of objects had been consolidated [9, 10]. Studies exploring the effects of perinatal acquired brain lesions on the development of visual perception are relatively rare [11–15]. These studies have described nonseverely impaired visual abilities following bilateral early damage to the primary visual cortex. Selective impairment of visual recognition and preserved visual memory are commonly reported findings. Performance on visual tasks and the different degrees of improvement at follow-up reveal the possible mechanisms of the visual system's developmental plasticity in child agnosia [11]. The study of visual object recognition in children with early brain damage might offer the opportunity of exploring the interaction between perception and memory in the course of development.

We investigated visual recognition and visual memory in three children with occipital lobe lesions (the striate cortex and to different degrees the dorsal and ventral streams) having occurred in early infancy and causing different patterns of perceptual impairment. We attempted to define their visual recognition deficits according to the classification of agnosia used in adults [1, 3]. These children also manifested visual memory impairment, which appeared to be modulated by the structural properties of objects. We also explored the time course of the visual memory impairment in two children we reexamined 2 years and 3.7 years from the initial assessment.

## 2. Methods

Between 2008 and 2009, each child underwent a neuropsychological testing battery, evaluating general cognitive abilities, language, visual perception, and visual memory. Two children (G. and R.) were reassessed for language, visual perception, and visual memory tasks between 2011 and 2012.

For tasks where normative data were not available, we assessed as controls two groups of age-matched children having normal or corrected to normal vision. Performances on the Navon task were compared to those of a group of 10 children (mean age = 11.97, SD = 1.72, 4F/6M) with no history of neurological disease (healthy controls). In order to compare the specificity of the effect of degraded visual perception on visual memory, we included a second group of four control subjects (mean age = 12.25, SD = 1.26) with unilateral early-onset brain lesions, normal general cognitive abilities, and no evidence of visual agnosia (neurological controls). These individuals characteristics were as follows: F. (male, 14 years, right handed) had left mesial temporal sclerosis and seizures; M. (male, 11 years, right handed) had right temporal polar lesion and was a candidate for epilepsy surgery; A. (female, 12 years, right handed) had left posterior quadrantic dysplasia; C. (male 12 years, right handed) had left temporal lobe dysplasia and was a candidate for epilepsy surgery. 

Informed consent was obtained before each assessment, in accordance with the Declaration of Helsinki and with the requirements of the Ethical committee of our Hospital.

## 3. Case Histories

Clinical characteristics of the three subjects are summarized in Table 1.

**Figure 1:**
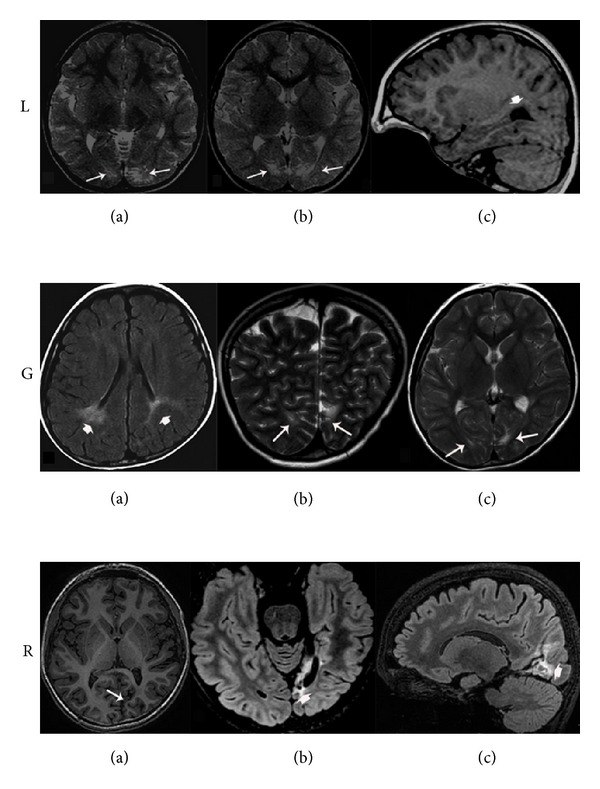
MRI scan for each patient. L.: (a), (b) Brain MRI (axial T2 and sagittal T1 weighted images) showing bilateral periventricular hyperintensities in the occipital lobes (white arrows) and dilatation of the occipital horn of the lateral ventricle (white arrowhead). G.: (a)–(c). Brain MRI (axial FLAIR, coronal and axial T2 weighted images) showing bilateral periventricular hyperintensities in the parietooccipital areas (see white arrowheads and arrows). R.: (a) Brain MRI (axial T1 weighted image) showing cortical-subcortical atrophy and abnormal cortical sulcation in the left temporooccipital area (white arrow). (b), (c). Brain MRI (axial and sagittal FLAIR weighted images) showing increased signal intensity in the left calcarine cortex and dilatation of the occipital horn of the left lateral ventricle (white arrowheads).

L., a 17-year-old right-handed boy, exhibited lethargy, hypotonia, and convulsions since the second day after birth. Hypoglycemia (plasma glucose level: 9 mg/L) was detected. He was treated with i.v. glucose, phenobarbital, and benzodiazepines. Recurrent hypoglycemia and convulsions occurred until 6 months of age, when he started exhibiting left hemiclonic seizures in euglycemia. A combination of vigabatrin and valproate was effective in controlling seizures. At 9 years of age he started to exhibit weekly drug-resistant seizures with unresponsiveness, head deviation to the left, hypotonia, and falls. Interictal EEG showed left temporoparietooccipital spikes and slow waves with contralateral spread. MRI revealed a linear hyperintense signal involving bilaterally the parietooccipital and calcarine sulci (Figure 1). Visual field testing revealed bilateral inferior quadrantanopia. When he was 11 years old he attracted our attention during clinical evaluation of epilepsy. Neuropsychological assessment, including visual recognition and visual memory testing, was conducted when he was 13 years old. After that year his family discontinued follow-up.

G., a 14-year-old right-handed boy, had experienced heart rate deceleration in the 36th week of pregnancy and was delivered by caesarean section. Apgar scores were 0–7 at 1 and 5 minutes after delivery. He had normal psychomotor development. At age 2, he experienced a right unilateral, prolonged seizure, and between age 6 and 8, three additional nocturnal seizures with right arm jerking. Since age 8 he exhibited diurnal seizures with initial visual hallucinations, pallor, cyanosis, right arm jerking with head, and eye deviation to the right. EEG showed bilateral asynchronous parietooccipital spikes. Brain MRI revealed high signal intensity in the left parietooccipital and calcarine sulci with atrophy of the left occipital lobe (Figure 1). Visual field testing revealed right inferior quadrantanopia. Carbamazepine treatment achieved complete seizure control. His last seizure occurred at age 9.5. Neuropsychological assessment, including visual recognition and visual memory testing, was performed when he was 10 years old and repeated at 13 years 7 months.

R., a 15-year-old right-handed boy who had an uneventful family and personal history, was diagnosed with right homonymous hemianopia at age 7. Three years later, seizures appeared, characterized by amaurosis and unresponsiveness. Brain MRI revealed an atrophic lesion involving the left mesial temporooccipital cortex (Figure 1). Carbamazepine was started with reduction of seizure frequency. From age 11 he experienced weekly episodes of head and eye deviation to the right, stiffening with subsequent fall and prolonged post-ictal aphasia. Antiepileptic drug treatment was ineffective. He progressively developed expressive language impairment and learning difficulties. A protocol for presurgical evaluation of epilepsy was started. Prolonged video-EEG recordings captured his habitual seizures which were accompanied by a left predominant ictal discharge. 

Functional MRI demonstrated left hemispheric dominance for language. Invasive EEG using subdural grids demonstrated that the seizure onset zone corresponded to the lesional area. At age 13.7, left temporal lobectomy was performed, sparing the superior (T1) and middle (T2) temporal gyri, and parietooccipital corticectomy. Histology revealed focal cortical dysplasia type IIId. Postoperative EEG was normal. He is seizure-free for 18 months after surgery. He developed mild expressive language impairment requiring speech therapy. Visual field defect and verbal memory impairment were unchanged in postsurgical evaluation and are documented in the Results section. Neuropsychological assessment, including visual recognition and visual memory testing, was conducted when he was 12 years old and repeated at the age of 14, four months after surgery.

## 4. Results

### 4.1. Neuropsychological Assessment at Baseline

This report is divided into two subsections. In this subsection we present data of the first neuropsychological testing administered to G., L, and R. In the next subsection we report data on follow-up evaluation of G. and R.

#### 4.1.1. Intelligence

General cognitive abilities were tested using the Wechsler Intelligence Scale for Children-Revised [16]. Each subject scored within average limits on the verbal scale and below average on the performance scale. L. and R. had Verbal IQs in the borderline range (VIQ = 74 and 77) and G. had a Verbal IQ in the average range (VIQ = 94). Performance IQs were mildly below average for L. and R. (PIQ = 64 and 67) and in the borderline range for G (PIQ = 74).

#### 4.1.2. Verbal Fluency

Spontaneous speech was fluent or relatively fluent with appropriate articulation, phonology, vocabulary, syntax, and prosody for each subject. Verbal fluency was within average range [17] for L. and G. for either phonemic (*z* = −0.05 and *z* = −1.5) or semantic (*z* = −1.65 and *z* = −1.03) condition; R. had a score below average on phonemic and on semantic fluency (*z* = −2.47, *z* = −1.90).

#### 4.1.3. Visual Discrimination

In a task similar to the Efron shape test [18], we presented black geometrical shapes on a white background, using a LCD monitor. In the first condition, ten squares with different sizes (from 2.6 to 6.2°) were presented in pairs, for 12 trials. Within a pair the size varied according to seven levels of difference (from 0.0 to 1.2°). In the second condition, a single shape (square of six sizes, 4.0 to 7.0° or rectangle of six dimensions, 3.25 × 3.5 to 6.5 × 9.0°) was presented, for 12 trials.

All children performed correctly at deciding if two squares were similar (L. = 100%, G. = 92%, R. = 83%,  *χ* ^2^(2) = 2.12,  *P* = .35) and if the shape corresponded to a square or a rectangle (L. = 92%, G. = 83%, R. = 75%;  *χ* ^2^(2) = 1.17,  *P* = .56). R. performance was slightly worse in the above task requiring fine-form discrimination.

In the shape discrimination task [17] children were asked to select in ten trials which of 9 abstract line drawings matched the target on the top of the sheet. All of them performed correctly (L. = 90%, G. = 80%, R. = 100%).

#### 4.1.4. Cancellation and Counting

In the visual search task L. correctly crossed 22/24 stars (92%, the two stars missed were one in the left and the other in the right part of the sheet), G. correctly cancelled 19/24 stars (79%, missed stars were not lateralized), and R. cancelled 19/24 stars (79%), missing stars towards the right side of the sheet, thus showing contralesional visuospatial hemineglect.

In the dot counting task, a number of black dots ranging from 3 to 19, with 3 to 7 mm distance from each other and 6 mm diameter, were printed on 10 white sheets. 

L. performed the task without errors (10/10), G. counted correctly 9/10 dots configurations, and R. counted correctly 6/10 dots configurations, showing difficulties in counting more than 12 dots per sheet.

#### 4.1.5. Naming

*Drawings*. L. correctly named 21/60 (35%, below average) drawings of the Boston Naming Test (BNT) [19, 20]. Errors were mainly visual (25%), semantic (13%), and “do not know” responses (18%). Visual errors were incorrect generalizations from a perceived or unperceived detail (e.g.: pretzel > *handcuffs, *wreath > *bow*, stilts > *ski*, unicorn > *horse*, palette > *paintbrush*).

G. correctly named 35/60 (58%, average) drawings on the BNT. Errors were mainly visual (16%), semantic (8%), and adequate circumlocutions (8%). Visual errors were incorrect generalizations from a perceived or unperceived detail (e.g.: knocker > *slingshot, *asparagus > *stick*, trellis > *ladder).*

R. correctly named 34/60 drawings (57%, below average). Errors were mainly visual (12%), semantic (12%), and “do not know” responses (8%).

G. and R. performed a tachistoscopic presentation of the BNT drawings 4–6 months later. Stimuli remained on the LCD screen for 500 msec. G. showed a slight increase in correct naming (40/60, 67%), while R. showed a relevant decrease (22/60, 37%) in total accuracy.

*Silhouettes*. Children were requested to identify 48 black drawings (24 living and 24 nonliving objects) on display until a first attempt at naming them. 

L. correctly named 23 silhouettes (48%) without differences in naming living (12/24) or nonliving objects (11/24). G. identified 41 silhouettes (85%; 22/24 living and 19/24 nonliving). R. identified 29 silhouettes (60%; 16/24 living and 13/24 nonliving).

*Overlapping Figures*. Children were presented 12 series of 4 geometrical figures and 12 series of 4 letters. The overlap concerned only one hidden figure while the remaining three had at least some uncovered contours.

L. correctly performed on 10/12 (83%) patterns of geometrical figures and 9/12 (75%) patterns of overlapping letters. G. correctly identified geometrical figures on 11/12 (92%) trials and letters on 11/12 (92%) trials. R. correctly identified geometrical figures on 10/12 (83%) trials and letters on 10/12 (83%) trials.

*Famous Faces*. Sixteen pictures of the best known Italian soccer players were presented on the LCD screen. All children were habitual soccer fans, knew the main teams, and collected stickers of the Italian national team.

L. recognized 1/16 (6%) football players showing a deficit in the recognition of famous faces. G. recognized 9/16 (56%) football players and R. 8/16 (50%).

#### 4.1.6. Copying

**Figure 2:**
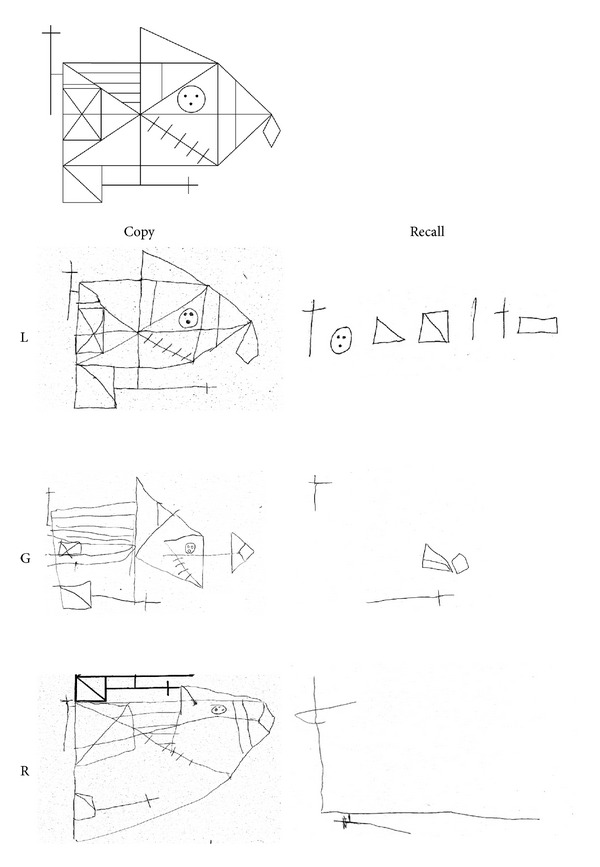
Rey's figure: copy and recall after a 15-minute delay performed by L., G., and R. The good copy realized by L. required a very slow process, beginning with the triangle-rectangle at the left top of the figure and adding segment by segment until the end. G. reproduced some details with general spatial contiguity. R. closed in on model and showed difficulties in drawing both details and global shape. All children had poor recall: L. could recall local details but failed to integrate them into an integrated whole, whereas G. and R. drawings were limited to very few details.

The Rey Figure [21] was used to assess drawing abilities in copying (Figure 2). L. realized a very good copy (score 31/36), but with a very slow process, beginning with the triangle-rectangle at the left bottom of the figure and adding segment by segment until the end, for about 20 minutes. G. had difficulties in copying the global structure and only reproduced some details with general spatial contiguity. He obtained a score of 12.5/36, below average. R. showed difficulties in this task with closing-in phenomenon and obtained a score of 16/36, below average.

#### 4.1.7. Reading

Each child was asked to perform two tasks taken from the Battery for the Evaluation of Dyslexia and Dysorthographia (DDE-2) [22]. In the first task they were requested to read four lists of words as quickly as they could, and in the second task to read three lists of nonwords.

G. performed at normal levels for time and accuracy. L. and R. were very slow in reading either words and nonwords. Their reading speed fell below the norm for children at the same school grade.

#### 4.1.8. Writing

All three children were able to write correctly words and sentences [22], spontaneously and under dictation.

#### 4.1.9. Navon Test

Target letters (S or T) were present at a global and/or a local level. The global letter subtended 3.276° × 3.66° of visual angle (in width and height, resp.), and each local letter subtended 0.56° × 0.48°. The interelement distance was 0.1°. 

There were two conditions: a global condition, in which the subject had to respond to global letters, ignoring local ones, and a local condition in which the subject had to respond to local letters, ignoring global ones. Three types of trials were presented: congruent (global letter was the same as the local letter), incongruent (global and local letters were different), or neutral (the target letter was associated with the letter X). The stimuli were presented on a PC running E-Prime software (Version 1.2) [23]. Participants responded via the keyboard, pressing one of the two keys assigned to the target letters. The order of the different conditions was balanced. There were 72 trials for each condition.  Table 2 shows error rates for L., G., R., and healthy controls for the global-local task.

A global interference effect, where local congruent letters produce a high proportion of accuracy than local incongruent, was observed for healthy control subjects (*χ* ^2^(2) = 21.27, *P* < .001). The reverse effect, local precedence, was not observed for healthy controls in the global condition (*χ* ^2^(2) = 2.71, *P* = .26). L., G. and R. exhibited an exaggerated global interference effect in the local condition (*χ* ^2^(2) = 9.15, *P* < .05). They also sustained a local interference effect in the global condition (*χ* ^2^(2) = 6.99, *P* < .05), although this effect emerged only for G., and R. accuracy rates. The contrast between the processing of local and global aspects of hierarchical forms demonstrated a strong bias to attend to global shape in the local condition for L., G., and R. However, the results for both G. and R. indicate also a bias to attend to local elements. These two children were generally impaired in their processing of hierarchical forms. This suggests that G. and R. find it difficult to attend to hierarchical forms mainly when there is competition of the local/global levels, than in trials in which competition is absent. 

#### 4.1.10. Verbal Memory

L. and G. obtained average scores (*z* = −.28 and *z* = .65) in paired-associated learning [17], whereas R. learned only few and simple associations after the three presentation of the same list of ten pairs of words. His score was below average (*z* = −2.66).

Incidental semantic memory was assessed presenting 20 animal names. Each child was requested to name the color of the animal but was not instructed to remember the items. At the end of the incidental learning phase, children were asked to report as many animals as they could. In the recall phase G. reported 10/20 animals, performing within average; L. and R. performed below average (5/20 and 6/20, resp.).

#### 4.1.11. Visual Memory

*Rey Figure Recall.* All children failed the recall of the complex figure (mean score = 3, SD = 1.32). L. could recall local details but failed to integrate them to the global structure, whereas G. and R. drawings were limited to very few details with no spatial relations with respect to the model (Figure 2).

Neurological control subjects recall was in the normal range (Mean score = 17.75, SD = 7.24).

**Figure 3:**
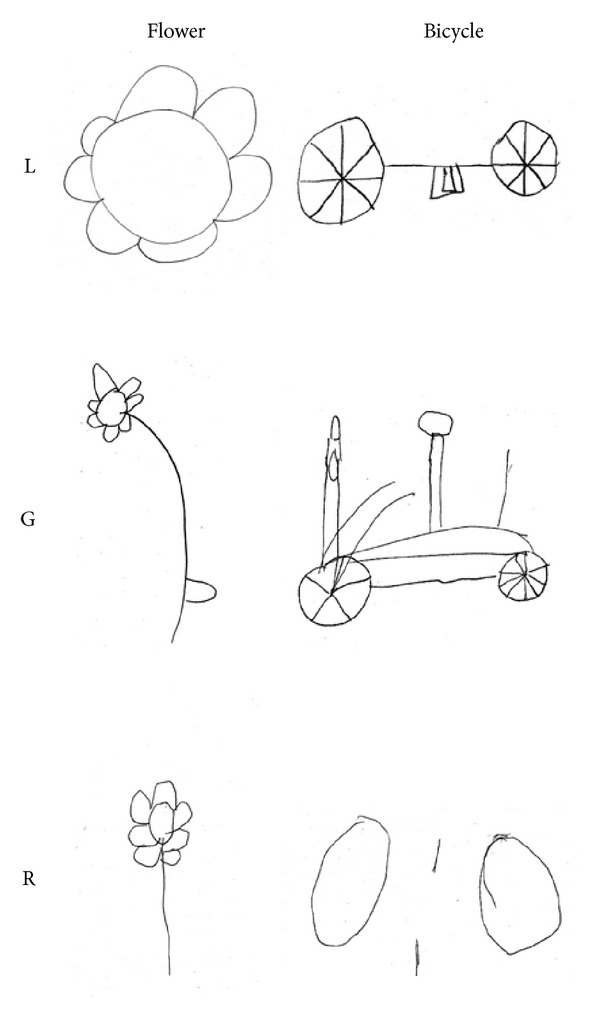
Examples of drawings from memory performed by L., G., and R. (a flower, a house, and a bicycle). Drawings were based on typical representations of objects. L. and R. limited their drawings of a bicycle to the emerging details (wheels).

*Drawing From Memory*. Subjects had to draw three objects: a flower, a house, and a bicycle (Figure 3). All children complained about the difficulties of drawing while accomplishing the task. They performed quite well in drawing a typical representation of a flower and a house. For the bicycle L. and R. omitted more visual properties, limiting their drawings to the emerging details (wheels). R. at the end of his task said “I would not ride that bike!”.

*Benton Visual Retention Test*. Children were presented 10 geometrical designs of the Benton Visual Retention Test, (BVRT) Part A [24] for 10 seconds and asked to reproduce them immediately afterwards. In this task the verbal component is important because subjects may remember the names of geometric figures without relying on stored visual representations to perform the task.

L. performed at average (8/10) and G. and R. were below average (4/10 for both). Neurological control subjects performed at average (Mean score = 8, SD = 0.82).

Following the same procedure used for the BVRT we administered two more visual memory tasks (stimulus images courtesy of Michael J. Tarr, Center for the Neural Basis of Cognition and Department of Psychology, Carnegie Mellon University, http://www.tarrlab.org/).

*Visual Retention of Objects.* Children were presented five drawings of objects, each on the center of a midsheet, selected for their high familiarity and for being composed of at least two different parts. They were *broom, boat, egg in eggcup, balloon*, and *bomb*. These objects may benefit from verbal rehearsal strategies for storage in memory. Each figure was assigned a value ranging from 0 to 2 depending upon the degree to which it was correctly drawn in local details, global form, and orientation.

**Figure 4:**
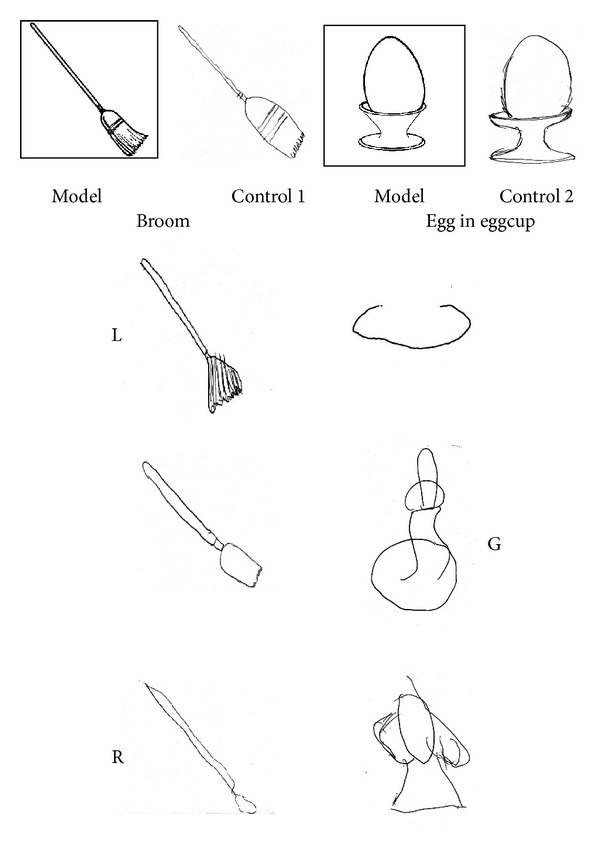
Two examples of immediate visual memory for objects performed by L., G., and R. The model is shown at the top, with the same object recalled by two neurological control subjects on the right. L., G., and R. had difficulties in reproducing the visual properties of each model and their drawings were limited at most to the typical representation of objects.

L. and G. obtained a score of 2/10 and R. scored 1/10 (mean = 1.67, SD = 0.58). Their performance was significantly worse than that observed in neurological control subjects (mean = 7.75, SD =.96; t(5) = 9.64, *P* < .001). See examples in Figure 4.

*Visual Retention of Abstract Drawings*. Children were presented 7 abstract designs which differed in their visual and spatial characteristics. These designs may not benefit from verbal rehearsal strategies to be stored in memory. Each figure was assigned a value of 0 to 2 depending upon the degree to which it was correctly drawn in local details, global form, and orientation.

**Figure 5:**
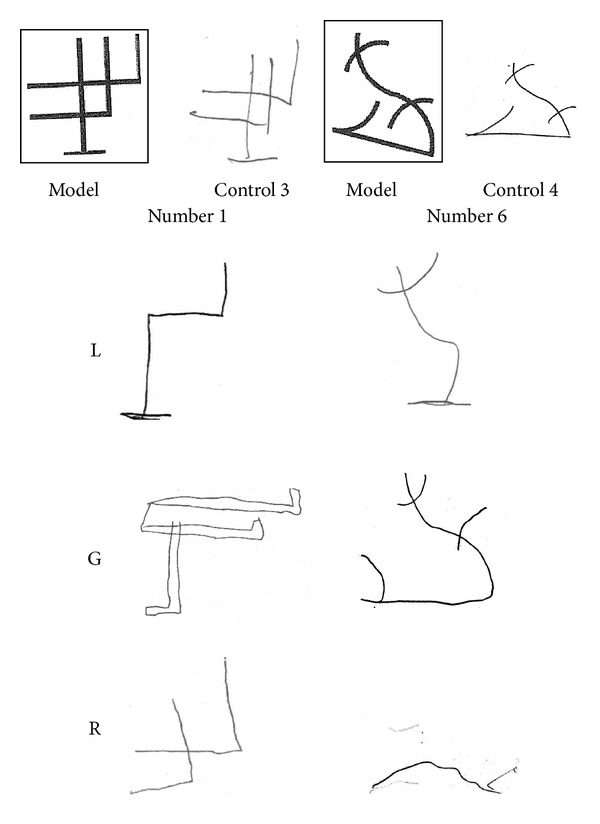
Two examples of immediate visual memory for abstract designs performed by L., G., and R. The model is shown at the top, with the same object recalled by two neurological control subjects on the right. All three children were unable to draw from memory a coherent pattern of a model that did not allow verbal rehearsal strategies.

Children were unable to draw from memory the abstract patterns (Figure 5), omitting or placing erroneously some parts (Mean score = 1.83, SD = .29). Neurological control subjects obtained a mean score of 9.25 (SD = .64; t(5) = 18.24, *P* < .001).

Although perceptual tasks were performed at different levels and degrees of impairment, each child revealed severe impairment in visual memory, slightly modulated by visual-semantic properties of recalled objects.  Table 3 summarizes the neuropsychological results for this section.

Each child performed relatively well on tests assessing basic aspects of visual perception. Their impairment at constructing a perceptual representation from vision and the consequent inability to copy or identify a drawing are consistent with apperceptive agnosia [3, 25]. Children evidenced qualitative differences in copying abilities and integration of shape elements into perceptual wholes. L. was impaired at integrating parts with whole shapes, and his performance is consistent with integrative agnosia: he performed correctly in the Efron test, reproduced an accurate copy of Rey's complex figure, had an abnormally strong bias to attend to global shape in the Navon task, though his performance was not facilitated by silhouettes in naming objects. R. impairment is consistent with visual-form agnosia: he was less accurate in the Efron test, his drawings were inaccurate, and he had an abnormally strong bias to attend to local and global elements in the Navon task. G. showed mild deficits in both integration and segmentation of complex visual objects.

### 4.2. Follow-Up for G. and R

G. and R. were reexamined after 3.7 years and 2 years, respectively, from the first evaluation. G. was 13 years old and R was 14 years old. R. completed the follow-up evaluation four months after neurosurgery.

#### 4.2.1. Boston Naming Test

G. correctly named 45/60 (75%) drawings, with a 17% improvement from the first evaluation. Errors were mainly visual and semantic. R. correctly named 9/40 drawings (22.5%), manifesting a relevant decrease (−34.5%) in performance. Errors were mainly “do not know” responses (30%) and adequate circumlocutions (27.5%). While the improvement in naming observed for G. might be interpreted as a normal developmental effect, R. impairment in naming was likely related to language impairment that occurred after surgery. 

#### 4.2.2. Copying: Rey Figure

**Figure 6:**
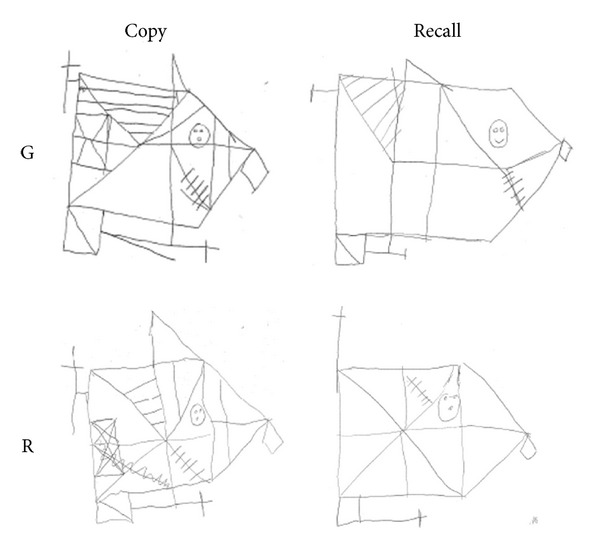
Rey's figure: Copy and Recall performed by G. and R. at follow up. A relevant improvement was observed in their drawings, both in the details and in their into a global shape, compared to the first assessment.

G. improved his abilities in copying the global and local features, obtaining a score of 23.5/36, now falling within average, with an increment of 46.8% on the previous score. Similarly, R. showed an improvement of 48.4% on the actual score (of 31/36, average), compared to the first assessment. These improvements (Figure 6) are not explained by practice effect (at least two years have passed since the first assessment) nor by a developmental effect (normative data show slight differences in scores from age 11 to adulthood).

#### 4.2.3. Visual Memory

*Rey Figure Recall.* G. increased his recall of the complex figure (12 versus 3.5, +70.8%), though his performance remained below average (*z* = −2.82). R. showed a greater improvement (18.5 versus 1.5, +91.9%) and his performance fell within average (*z* = −1.15), with no difference from neurological controls (Mean score = 17.75, SD = 7.24). See Figure 6.

*Benton Visual Retention Test.* G. and R. were still below average (3/10 and 6/10, resp.), showing no improvement in the recall of geometrical drawings. 

**Figure 7:**
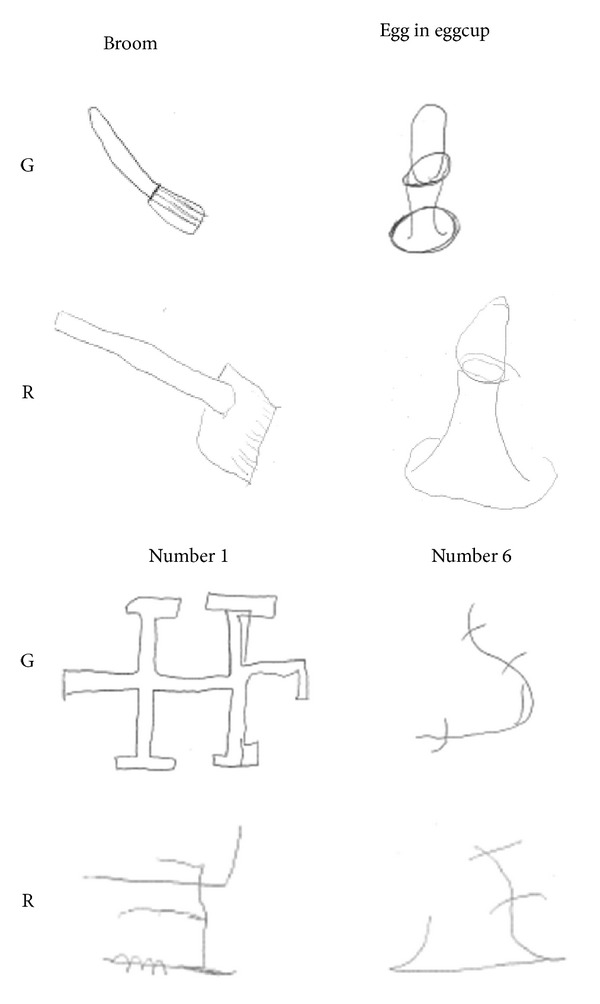
Examples of immediate visual memory for objects and abstract drawings performed by G. and R. at follow-up. Both children improved the quality of their drawings from memory by adding more details in an integrated whole, compared to the previous testing.

*Visual Retention of Objects*. G. obtained a score of 7/10, with a five-point improvement from the initial assessment (2/10). R. reported a score of 2/10, one point higher than previously observed (1/10). Two examples are illustrated in Figure 7. 

*Visual Retention of Abstract Drawings.* Both children improved the quality of their drawings from memory for the seven abstract patterns (Figure 7): G. with a score from 1 to 5/14 and R. from 0 to 4/14, compared to the previous assessment. However, their scores were still below the average observed in neurological control subjects.

These results demonstrated a global improvement in perceptual and memorial processes over time. There was a general increase in performance at follow-up from the previous assessment in almost all tasks, with the exception of the Benton Visual Retention Test. Improvement was higher in memory tasks relying on the storage of structural properties of objects. When memory was based only on pure visual properties this incremental effect was limited. However, R. showed a deterioration in naming and no change in visual retention of objects. 

## 5. Discussion

We are presenting behavioural data of three children with visual agnosia and visual memory impairment, caused by brain lesions of the visual cortex which occurred in early infancy.

Children exhibited relatively normal sensory and semantic memory functioning, with several difficulties in object recognition. They also exhibited deficits in copying, naming drawings, and integrating object features. We classified their impairments in two types of apperceptive agnosia. R. exhibited visual-form agnosia, L. integrative agnosia, and G. mild difficulties in segmentation and integration processes that share some characteristics with that observed in L. Resemblance between these findings and those described in adult agnosics [3, 26] provides further evidence that the associations between specific impairment and site of lesion are concordant in both adults and children [27].

Reports on childhood agnosia are relatively rare. These include both acquired cases of early [11, 14] and late onset [9, 10, 12] origin. Kiper and collaborators [11] described two patients (one of them was evaluated during childhood) with bilateral perinatal lesions of the primary visual cortex. They exhibited recognition deficits, which were classified as apperceptive agnosia at different degrees of severity, and manifested different levels of adaptive plasticity over time [11]. Amicuzi and collaborators [14] reported a 4.6-year-old girl with bilateral occipital damage and visual agnosia who manifested selective deficit of figure-ground segregation, impaired visual recognition, and abnormal moving through space. Both case reports emphasized the “fuzzy boundaries” [28] between visual-form agnosia and integrative agnosia, as far as children's behavior and domain-specific functions are concerned. Compared to adults, the differences between the two types of agnosia are even less distinguishable, possibly because developmental neurocognitive mechanisms are flexible and adaptive [27]. Nonetheless, these studies indicate that the core deficit of apperceptive agnosia can indeed be identified in children with acquired lesions of the visual cortex. 

In previously reported observations of childhood agnosia, no evidence of visual memory impairment, as assessed by standard tests, was uncovered [11, 14]. We observed degraded object recognition and associated deficits of visual memory in apperceptive agnosia. All three children exhibited impaired visual memory and poor drawing from memory with, however, some evidence that residual visual knowledge might be built up relying on the structural properties of objects. 

As interpreted in the longitudinal investigation of the adult agnosic H.J.A., when perceptual inputs are degraded, as in apperceptive agnosia, visual memory may decline, due to impaired fine-tuning of the system to the visual properties of objects [8]. Conversely, we observed a longitudinal improvement of visual memory, modulated by object properties, suggesting that underlying developmental mechanisms may act from infancy to late childhood. The impairment was more severe for visual-spatial compared to visual-object properties, showing a dissociation in processing of visual representations. These findings are consistent with the hypothesis of a common representation of an image that is processed in the visual buffer [29]. At this level, a selective damage may occur to the spatial and object mechanisms that act on the image [30]. Developmental processes might selectively adapt and modulate these mechanisms to the neuroanatomical substrate for image construction. Indeed, the improvement we observed in R. and G. visual memory at follow-up was more apparent when the task relied on the structural properties of objects and less so when memory concerned only pure visual-spatial properties of objects. These preliminary findings have important implications for understanding the development and construction of visual representations. More longitudinal studies are needed to shed light on the dynamic interaction between visual perception and visual memory during development, when the occipital cortex is damaged from birth.

## 6. Conclusions

Impairments of visual recognition are relatively overlooked in children, nevertheless they have a crucial impact on learning as well as mobility and independent living.

Our findings indicate that impaired processing of visual images in childhood agnosia results in impoverished visual memories. During the course of development the dynamic interaction between perception and memory might modulate the long-term construction of visual representations, resulting in less severe impairment.

As suggested by previous reports, developmental brain plasticity and compensatory behavioral adaptations allow the emergence of surprising abilities in daily life [12]. Therefore, an early diagnosis of visual agnosia is crucial for planning specific rehabilitation interventions that may foster the progressive adaptation leading to a developmental process that is unique in a child with brain damage. 

Early neuropsychological rehabilitation in childhood visual agnosia should train visual and multimodal environmental exploration, improving recognition and memory through the processes of adaptation and compensation that are the hallmark of development following perinatal neural pathology.

## Figures and Tables

**Table 1:** Clinical characteristics for each child*.

	L.	G.	R.
Age (years)	12	10	12
(i) at seizure onset/ (ii) at last seizure	0,2/6; 9/still present	6/9,5	6/9,5
Birth and delivery	Hypoglycemia (neonatal convulsions)	Perinatal hypoxia	Uneventful
Visual field	Bilateral inferior quadrantanopia	Right inferior quadrantanopia	Right hemianopia
Interictal EEG abnormalities	Left temporoparietooccipital spikes and slow waves with contralateral spread	Bilateral asynchronous temporoparietooccipital spikes and diffuse spike and wave discharges	Diffuse spikes and waves while awake and diffuse polyspike discharges during sleep, left predominance
Ictal EEG	NA	NA	Diffuse spike discharge with left predominance
Seizure semiology	Unresponsiveness, head deviation to the left, hypotonia, and falls	(a) During sleep: right arm jerks and ocular revulsion (b) While awake: visual hallucinations, pallor, cyanosis, right arm jerks, and head and eye deviation to the right	(a) Amaurosis, abnormal eye movement, and unresponsiveness; (b) Head and eye deviation to the right, stiffening with frequent falls; postictal aphasia
Treatment	VPA + ESM + TPM	CBZ	LTG + OxCBZ

*NA: not available; CBZ: carbamazepine; ESM: ethosuccimide; LTG: lamotrigine; OxCBZ: oxcarbazepine; TPM: topiramate; VPA: valproic acid.

**Table 2:** Percentage of error for responses in the Navon task by local and global conditions.

	Local			Global		
	Congruent	Incongruent	Neutral	Congruent	Incongruent	Neutral
Healthy controls	1,4	8,8	1,4	1,4	3,7	3,7
L.	4,2	20,8	16,7	4,2	4,2	8,3
G.	8,3	20,8	4,2	0	20,8	0
R.	4,2	25	12,5	4,2	16,7	8,3

**Table 3:** Performance of each agnosic child on perception and memory tasks.

	L.	G.	R.
Efron tasks	Not impaired	Not impaired	Not impaired, some errors
Shape discrimination	Not impaired	Not impaired	Not impaired
Dot counting	Not impaired	Not impaired	Impaired
Star cancellation	Not impaired	Impaired, not lateralized misses	Impaired, right misses
Boston naming test	Below average, visual errors	Average, visual errors	Below average, visual and semantic errors
BNT at 500 ms	—	12.5% improvement	35% Worsening
Silhouettes	Impaired No differences for living/nonliving	Not impaired Living objects better	Intermediate No differences for living/nonliving
Overlapping figures	Slightly impaired	Not impaired	Slightly impaired
Famous faces	Impaired	Not impaired	Not impaired
Rey figure copying	Average, but very slow process	Impaired	Impaired
Reading	Slow	Normal	Slow
Writing	Relatively normal	Relatively normal	Relatively normal
Navon task	Global effect exaggerated	Global and local effect exaggerated	Global and local effect exaggerated
Verbal memory	Not impaired	Not impaired	Impaired
Rey figure memory	Impaired	Impaired	Impaired
Immediate visual memory			
(i) Benton VRT	Impaired	Impaired	Impaired
(ii) Objects	Impaired	Impaired	Impaired
(iii) Abstract designs	Impaired	Impaired	Impaired
